# Milk

**Published:** 1861-03

**Authors:** 


**MILK.**—Prof. Reese, of Union Square, who is one of the best medical scholars in the United States, says, in a late issue, of the *Rockland County and New-Jersey Milk Association*, 146 East-Tenth street, near Broadway, New-York: "We have taken great interest in this movement, recommended and used the milk thus furnished, and many of our patients' children are thriving under that furnished by a single cow, and which is sold separately. If the Company continue to perform what they promise, their patronage may be indefinitely extended, and they will be public benefactors." We add our testimony to the same effect.

